# Knowledge, Attitudes, and Self‐Reported Tetanus‐Toxoid‐Containing Vaccine Uptake Among Women Aged 15–44 Years in Ifakara Town, Tanzania: A Community‐Based Cross‐Sectional Study

**DOI:** 10.1002/hsr2.73262

**Published:** 2026-09-14

**Authors:** Dorothea R. Mhina, Zacharia L. Laizer, Onesmo Tisiani Hilari, Byamungu Zephrine Petro, Samweli Y. Bahati, Reuben S. Maghembe

**Affiliations:** ^1^ Department of Microbiology and Immunology, Faculty of Medicine St. Francis University College of Health and Allied Sciences Ifakara Tanzania; ^2^ Department of Microbiology and Immunology, School of Diagnostic Medicine Muhimbili University of Health and Allied Sciences Dar es Salaam Tanzania; ^3^ Department of Medical Laboratory Sciences, Institute of Allied Health Sciences St. Francis University College of Health and Allied Sciences Ifakara Tanzania; ^4^ Department of Omics and Computational Biology AfroBiomics Co. Ltd. Dar es Salaam Tanzania

**Keywords:** access barriers, dose completion, health literacy, healthcare‐provider communication, maternal immunization, parity, reproductive health

## Abstract

**Background and Aims:**

Tetanus‐toxoid‐containing vaccines protect mothers and newborns, but cumulative uptake may decline at higher dose thresholds. We assessed knowledge, attitudes, self‐reported tetanus‐toxoid‐containing vaccine uptake, and factors associated with receipt of at least three doses among women aged 15–44 years in Ifakara Town, Tanzania.

**Methods:**

A community‐based analytical cross‐sectional study included 383 women aged 15–44 years; electronic records were submitted between September 14, 2025, and January 6, 2026. Participants were selected through multistage sampling and completed an interviewer‐administered questionnaire. The primary outcome was self‐reported receipt of at least three tetanus‐toxoid‐containing vaccine doses. Participant‐level data were reanalyzed using multivariable logistic regression. The parity‐adjusted complete‐case model included 382 women; Firth bias‐reduced regression assessed sparse‐data sensitivity.

**Results:**

Almost all participants had heard of tetanus vaccination (97.7%) and reported ever receiving it (97.4%). Self‐reported uptake was 80.7% for at least two doses, 66.6% for at least three doses, 38.4% for at least four doses, and 24.5% for five doses. Good knowledge was reported for 66.1%, and 74.4% had a positive attitude score. In the parity‐adjusted model, the odds of reporting at least three doses were higher for ages 26–35 years (aOR 2.89, 95% CI 1.34–6.20) and 36–44 years (aOR 5.89, 95% CI 1.66–20.96), healthcare‐provider information (aOR 2.06, 95% CI 1.07–3.97), and parous status (aOR 15.94, 95% CI 6.84–37.14). Education, knowledge, and reported access barriers were not independently associated after parity adjustment.

**Conclusion:**

Initial contact with tetanus vaccination services was high, but self‐reported uptake declined at higher dose thresholds. Age and parity probably reflect cumulative opportunities for vaccination, rather than causal effects. Dose‐history review, provider‐led counseling, and access support may improve cumulative uptake.

**Abbreviations**
ANC: antenatal care
aOR: adjusted odds ratio
CI: confidence interval
KAP: knowledge, attitudes, and practices
MNTE: maternal and neonatal tetanus elimination
OR: odds ratio
SFUCHAS: St. Francis University College of Health and Allied Sciences
SPSS: Statistical Package for the Social Sciences
TT: Tetanus toxoid
TTCV: Tetanus‐toxoid‐containing vaccine
WHO: World Health Organization

## 1 Introduction

Tetanus is a severe vaccine‐preventable disease caused by Clostridium tetani, an environmental spore‐forming bacterium that produces tetanospasmin, a neurotoxin responsible for the clinical manifestations of the disease [1, 2]. Because tetanus spores persist in the environment and infection does not reliably confer immunity, prevention depends on active immunization with tetanus‐toxoid‐containing vaccines, clean delivery practices, and hygienic umbilical cord care [2, 3]. Women of reproductive age are a priority group because adequate immunization before or during pregnancy protects mothers and contributes to the protection of newborns during the early neonatal period [4, 5].

Global progress toward maternal and neonatal tetanus elimination has been substantial, but the remaining risk is concentrated in settings where coverage with tetanus‐toxoid‐containing vaccines, skilled birth attendance, and clean delivery practices is incomplete [2, 3]. In sub‐Saharan Africa, coverage varies across and within countries, reflecting differences in health‐service access, maternal education, household resources, geographic barriers, antenatal‐care attendance, and health information exposure [4, 6, 7]. In Tanzania, tetanus vaccination is delivered mainly through maternal and child health services, yet local patterns of cumulative uptake, information exposure, and access barriers may differ between urban, semi‐urban, and rural‐linked communities [8, 9].

Knowledge, attitudes, and vaccination practices are useful behavioral dimensions for understanding maternal immunization, but they should not be interpreted as a simple linear pathway from awareness to uptake. Knowledge may include awareness of tetanus, recognition of vaccine benefits, understanding of vaccination during pregnancy, and knowledge of dose schedules. Attitudes may include perceived importance, safety, trust in providers, and acceptability. Practices include vaccine‐seeking behavior, recalled doses received, vaccination site, and response to barriers [10, 11]. Women may receive vaccines through antenatal‐care contact or provider recommendation even when detailed schedule knowledge remains incomplete, making cumulative uptake thresholds important descriptive outcomes [12, 13].

Ifakara Town, in Kilombero District, Morogoro Region, Tanzania, serves a semi‐urban population and surrounding rural‐linked communities. It is also a local health‐service hub, with public and private facilities providing maternal and child health services. These characteristics make Ifakara a relevant setting for examining whether high vaccine contact is accompanied by higher cumulative uptake and whether information sources and access barriers shape reported uptake.

This study assessed knowledge, attitudes, and self‐reported uptake of tetanus‐toxoid‐containing vaccination among women aged 15–44 years in Ifakara Town, Tanzania, and examined sociodemographic, informational, and access‐related factors associated with self‐reported receipt of at least three doses. No directional hypothesis was prespecified.

## 2 Methods

### 2.1 Study Design and Setting

A community‐based analytical cross‐sectional study was conducted in Ifakara Town, Kilombero District, Morogoro Region, southeastern Tanzania. The fieldwork was done during September–October 2025. However, the available Kobo export records submission timestamps from September 14, 2025, through January 6, 2026, and contain no separate interview‐date variable; therefore, the precise interview period could not be verified. Ifakara is located in the Kilombero Valley at approximately 8.1333° S and 36.6833° E. According to the 2022 Tanzania Population and Housing Census, Kilombero District had a population of approximately 617,000 people, with many residents living in rural or semi‐rural communities [14].

Ifakara Town functions as an administrative, commercial, educational, and health‐service center for Kilombero District. The population includes women with heterogeneous socioeconomic, educational, and healthcare‐access characteristics. Maternal and child health services, including antenatal care and immunization services, are provided through public and private facilities in and around the town. This setting was therefore appropriate for assessing tetanus‐toxoid‐containing vaccination knowledge, attitudes, uptake, and access barriers among women of reproductive age.

### 2.2 Study Population

The study population comprised women of reproductive age residing in Ifakara Town during the study period. Eligible participants were women aged 15–44 years who had lived in Ifakara Town for at least 6 months and were able and willing to provide informed consent. Women who were not permanent residents, severely ill at the time of data collection, or had cognitive impairment affecting informed consent were excluded.

### 2.3 Sample Size and Sampling Procedure

The target sample size was calculated using Cochran's formula for cross‐sectional studies, assuming a 95% confidence level, 5% margin of error, and an expected tetanus‐toxoid‐containing vaccination uptake prevalence of 51.5% from a sub‐Saharan African pooled estimate [4]. This yielded a target sample size of 384 participants. The available dataset contained 383 participant records and was used as the analytic sample. The surviving study records did not retain the numbers approached, screened, eligible, declining, excluded, or incomplete; a detailed recruitment flow therefore could not be reconstructed. A multistage sampling approach was applied. First, streets or villages within Ifakara Town were listed and selected by simple random sampling. Second, households within selected areas were selected systematically. Third, eligible women in selected households were identified; where more than one eligible woman was present, one was selected using a simple random method.

### 2.4 Data Collection Tool and Procedures

Data were collected using a structured interviewer‐administered questionnaire. The questionnaire captured sociodemographic characteristics, knowledge about tetanus and tetanus‐toxoid‐containing vaccination, attitudes toward vaccination using Likert‐scale items, self‐reported vaccination history, usual information sources, vaccination sites, and perceived access barriers (Supporting file 1). The tool was prepared in English, translated into Kiswahili, and back‐translated to check consistency. Data collectors were instructed to use the Kiswahili version during interviews to support standardized administration.

### 2.5 Measurement of Variables

Because the original scoring syntax was unavailable, knowledge was reconstructed for this revision from five binary components: having heard of tetanus vaccination; selecting prevention of tetanus; selecting prevention of neonatal tetanus; identifying vaccination during pregnancy as safe; and rating the vaccine as very or somewhat effective. Each correct or favorable response scored 1 (range 0–5); scores of 0–1, 2–3, and 4–5 were classified as poor, moderate, and good, respectively. The dose‐number knowledge item was excluded because the questionnaire omitted five doses as a response option. Ten attitude items were scored from 1 (strongly disagree) to 4 (strongly agree); the negatively worded concern‐about‐side‐effects item was reverse‐scored. Scores of 29–40 were classified as positive and 10–28 as neutral/non‐positive (Cronbach's alpha = 0.649). The primary outcome was self‐reported receipt of at least three doses. Because the form lacked a separate four‐dose option, the raw response “More than 3” was operationally classified as at least four doses; the response “5” was classified as five doses. These recalled thresholds were not treated as verified evidence of complete lifetime protection.

### 2.6 Statistical Analysis

The original data were coded in SPSS version 27 and independently reanalyzed for this revision using a reproducible Python 3.12.13 script with pandas 2.2.3, NumPy 2.3.5, and SciPy 1.17.0. Categorical variables were summarized as frequencies and percentages. Bivariable logistic regression estimated crude odds ratios. The main multivariable logistic regression model included age group, education level, knowledge level, healthcare‐provider information source, any reported access barrier, and binary parity (parous vs. nulliparous). The ≥ 3‐dose outcome and candidate predictors followed the manuscript's original analytic focus; parity adjustment, Firth regression, and the education‐composition assessment were revision‐stage sensitivity or explanatory analyses. One participant with missing parity was excluded from this complete‐case model (*n* = 382); no other model variable was missing, and no values were imputed. Maximum‐likelihood adjusted odds ratios (aORs), 95% Wald CIs, and two‐sided *p* values are reported. Firth bias‐reduced logistic regression was used as a sparse‐data sensitivity analysis. Model discrimination, calibration error, and multicollinearity were assessed using the area under the receiver operating characteristic curve, Brier score, and variance inflation factors. Sampling‐unit identifiers were unavailable, so ordinary rather than design‐adjusted standard errors were used. *p* < 0.05 denoted statistical significance, interpreted with effect size and precision. Associations were interpreted as noncausal. Additional methodological and analytical materials are provided in the Supporting File 1 and Supporting Information 1.

### 2.7 Ethical Considerations

Ethical approval was granted by the SFUCHAS Research Ethics Review Committee (reference SFU/Re. Pub/Eth. App/Vol.1/4; September 2, 2025); the approval covered women aged 15–44 years and was valid for 1 year. Ifakara Town Council granted research permission (reference IFTC/E.10/80 VOL V/118; 12 September 2025). Participants were informed about the study purpose, voluntary participation, confidentiality, anonymity, and their right to withdraw. The study report records written informed consent from all participants. The available documentation contains a general participant consent form but does not separately describe guardian permission, adolescent assent, an ethics waiver, or an emancipated‐minor provision for the 14 participants aged 16–17 years. No direct personal identifiers were retained in the analytic dataset used for reanalysis.

### 2.8 Reporting Guideline

This manuscript was prepared in accordance with the STROBE reporting guideline for observational cross‐sectional studies.

## 3 Results

### 3.1 Participant Characteristics

A total of 383 women were included. Participants were aged 15–25 years (151/383, 39.4%), 26–35 years (166/383, 43.3%), or 36–44 years (66/383, 17.2%). The raw parity responses were 0 children for 107 (27.9%), 1–3 children for 228 (59.5%), and “More than 4” for 47 (12.3%); one value was missing. Marital status was nearly evenly distributed between single women (175/383, 45.7%) and married women (174/383, 45.4%). Most participants had secondary education (235/383, 61.4%). Peasants were the largest occupational group (124/383, 32.4%), followed by self‐employed women (114/383, 29.8%) and students (85/383, 22.2%) (Table 1).

**Table 1:** Sociodemographic characteristics of study participants.

Characteristic	Category	Frequency, *n*	Percentage, %
Age group	15–25 years	151	39.4
	26–35 years	166	43.3
	36–44 years	66	17.2
Parity	0 children	107	27.9
	1–3 children	228	59.5
	More than 4 (as recorded)	47	12.3
	Missing	1	0.3
Marital status	Single	175	45.7
	Married	174	45.4
	Divorced/widowed	34	8.9
Education	Primary or below	94	24.5
	Secondary	235	61.4
	Tertiary	54	14.1
Occupation	Peasant	124	32.4
	Self‐employed	114	29.8
	Student	85	22.2
	Formally employed	37	9.7
	Unemployed	23	6.0

*Note:* The highest parity category is reported verbatim from the raw data export; a four‐child response option was not available in that field.

### 3.2 Knowledge, Attitudes, and Vaccination Uptake

Nearly all participants had heard of tetanus toxoid vaccination (374/383, 97.7%). The reconstructed knowledge score classified 253 participants (66.1%) as good, 126 (32.9%) as moderate, and 4 (1.0%) as poor. After reverse‐scoring the concern‐about‐side‐effects item, 285 participants (74.4%) had a positive attitude score and 98 (25.6%) had a neutral/non‐positive score; internal consistency was modest (Cronbach's alpha = 0.649) (Supporting Information 1).

**Figure 1:**
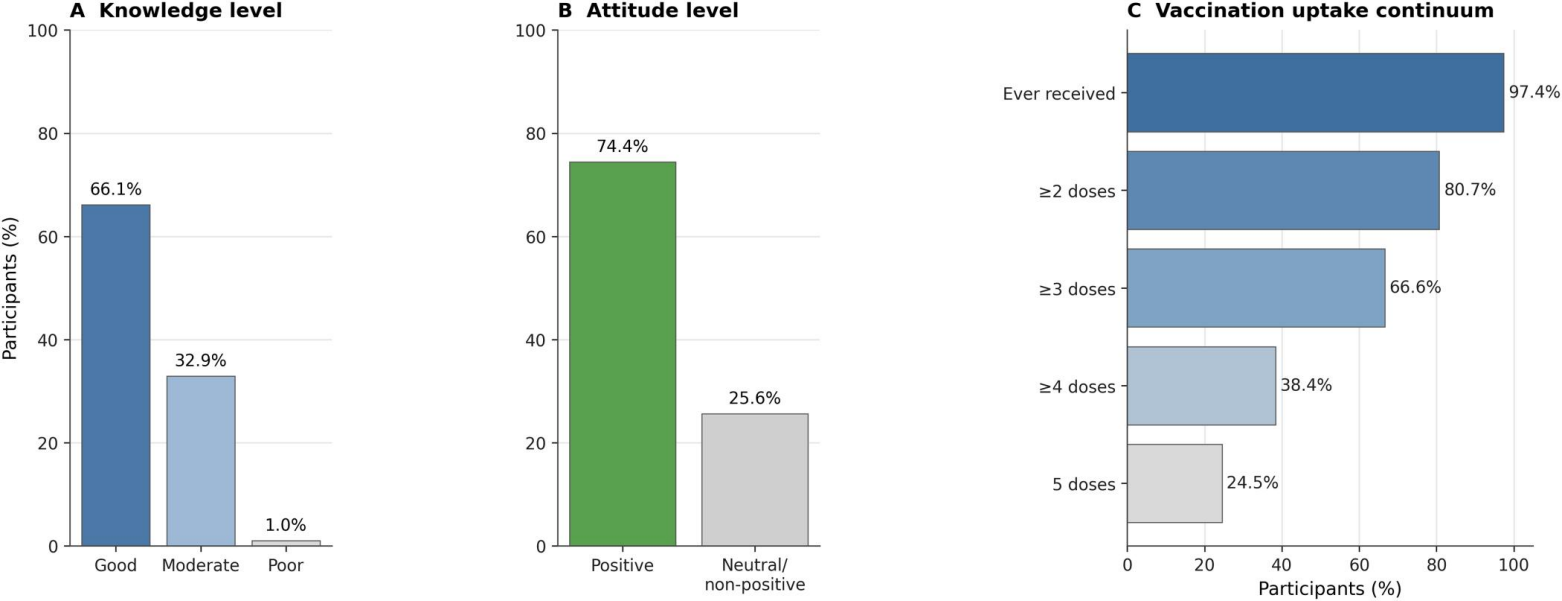
Knowledge, attitudes, and self‐reported tetanus vaccination uptake among women aged 15–44 years in Ifakara Town, Tanzania. (A) Reconstructed knowledge level. (B) Ten‐item attitude classification after reverse‐scoring the concern‐about‐side‐effects item. (C) Self‐reported uptake from ever receiving tetanus vaccination through cumulative dose thresholds. Percentages use the analytic sample of 383 participants.

Overall, 373 participants (97.4%) reported ever receiving tetanus toxoid vaccination. Reported uptake was 309/383 (80.7%) for at least two doses, 255/383 (66.6%) for at least three doses, 147/383 (38.4%) for at least four doses, and 94/383 (24.5%) for five doses (Figure 1).

### 3.3 Sources of Information, Vaccination Sites, and Perceived Barriers

Family and friends were the most common usual source of tetanus vaccination information (275/383, 71.8%), followed by healthcare providers (223/383, 58.2%). Public health facilities were the most frequently reported vaccination site (302/383, 78.9%), followed by hospitals (142/383, 37.1%), private clinics (88/383, 23.0%), and community programs (19/383, 5.0%). Overall, 136 participants (35.5%) reported at least one barrier to accessing tetanus vaccination. The most commonly reported barriers were cost (91/383, 23.8%), lack of information (54/383, 14.1%), transportation challenges (39/383, 10.2%), and fear of side effects (26/383, 6.8%) (Table 2).

**Table 2:** Information sources, vaccination sites, and perceived barriers.

Domain	Response category	Frequency, *n*	Percentage, %
Usual information source	Family/friends	275	71.8
	Healthcare provider	223	58.2
	Media	25	6.5
	Community programs	19	5.0
Vaccination site	Public health facility	302	78.9
	Hospital	142	37.1
	Private clinic	88	23.0
	Community program	19	5.0
Any barrier	Yes	136	35.5
Specific barriers	Cost	91	23.8
	Lack of information	54	14.1
	Transportation	39	10.2
	Fear of side effects	26	6.8

*Note:* Multiple responses were allowed for information sources, vaccination sites, and specific barriers; therefore, percentages may exceed 100% within each domain.

### 3.4 Factors Associated With Receipt of at Least Three Tetanus Vaccine Doses

**Figure 2:**
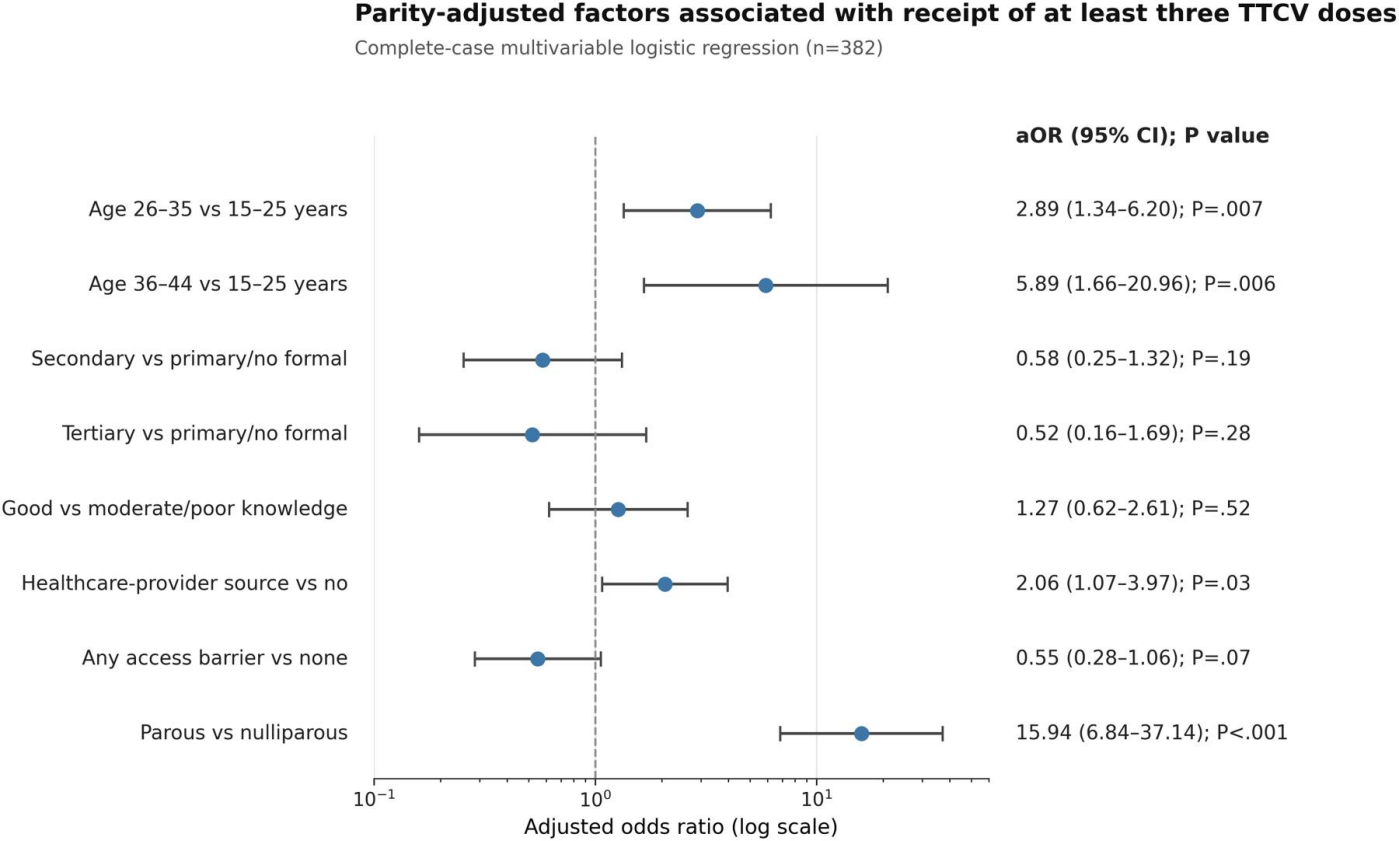
Parity‐adjusted factors associated with self‐reported receipt of at least three tetanus‐toxoid‐containing vaccine doses among women aged 15–44 years in Ifakara Town, Tanzania. The forest plot shows maximum‐likelihood aORs and 95% CIs from the complete‐case multivariable model (*n* = 382), which included age group, education level, knowledge level, healthcare‐provider information source, reported access barriers, and binary parity. The vertical line indicates an aOR of 1.0. Full crude, adjusted, diagnostic, and Firth sensitivity results are provided in Supporting Information 1.

Self‐reported receipt of at least three doses was reported by 49/151 (32.5%) women aged 15–25 years, 144/166 (86.7%) aged 26–35 years, and 62/66 (93.9%) aged 36–44 years. In the parity‐adjusted complete‐case model (*n* = 382), age 26–35 years (aOR 2.89, 95% CI 1.34–6.20; *p* = 0.007), age 36–44 years (aOR 5.89, 95% CI 1.66–20.96; *p* = 0.006), healthcare‐provider information (aOR 2.06, 95% CI 1.07–3.97; *p* = 0.03), and parous status (aOR 15.94, 95% CI 6.84–37.14; *p* < 0.001) were associated with higher odds of the outcome. Secondary education (aOR 0.58, 95% CI 0.25–1.32; *p* = 0.19), tertiary education (aOR 0.52, 95% CI 0.16–1.69; *p* = 0.28), good knowledge (aOR 1.27, 95% CI 0.62–2.61; *p* = 0.52), and any access barrier (aOR 0.55, 95% CI 0.28–1.06; *p* = 0.07) were not independently associated. Firth estimates were similar (Figure 2; Supporting Information 1).

The apparent inverse education association in the reduced model was attenuated after parity adjustment. Secondary‐educated participants were more often aged 15–25 years than those with primary/no formal education (48.1% vs. 28.7%) and were more often nulliparous (36.2% vs. 10.6%), supporting confounding by cumulative vaccination opportunity.

## 4 Discussion

This community‐based cross‐sectional study found high awareness and high self‐reported contact with tetanus‐toxoid‐containing vaccination among women aged 15–44 years in Ifakara Town. Uptake was high at the ≥ 2‐dose and ≥ 3‐dose thresholds but declined markedly at higher thresholds. Reanalysis of the participant‐level data corrected four crude odds ratios and showed that age, healthcare‐provider information, and parity, not education or the composite knowledge score, were independently associated with the ≥ 3‐dose outcome. The principal contribution remains the distinction between initial vaccine contact and recalled cumulative uptake rather than verified schedule completion or individual protection [2, 3].

Knowledge and attitude scores were generally favorable under the reconstructed rules, but these composites require caution. The dose‐knowledge question omitted five doses and was excluded. The attitude classification required reverse‐scoring one negatively worded item and had modest internal consistency (Cronbach's alpha = 0.649). The findings should therefore be interpreted as operational summaries of selected responses, not validated measures of complete schedule knowledge or a single latent attitude construct. Future instruments should include all locally applicable dose options and undergo cognitive and psychometric validation [8, 13].

The dose‐threshold pattern provides the clearest public‐health signal. Almost all participants reported ever receiving tetanus vaccination, and four‐fifths reported at least two doses, but fewer than two‐fifths reported at least four doses, and one‐quarter reported five doses. These are recalled cumulative thresholds, not verified completion. WHO life‐course guidance aims for six tetanus‐toxoid‐containing vaccine doses, while pregnancy and catch‐up schedules depend on reliable prior vaccination history [2]. Similar dose drop‐off has been reported in Nigeria and Ethiopia, although definitions and populations differ [12, 15]. The programmatic implication is to strengthen dose‐history review, documentation, counseling, and follow‐up.

Older age remained associated with self‐reported receipt of at least three doses after adjustment for parity, but its estimates were substantially attenuated relative to the reduced model. Older women have had more time, pregnancies, antenatal‐care contacts, and opportunities for vaccination. The wide CI in the oldest group and the non‐causal design argue against interpreting age itself as a mechanism [4, 16].

Good knowledge and reported access barriers were associated with uptake in the reduced model, but their CIs crossed 1 after parity adjustment. This attenuation does not make knowledge or access unimportant; it shows that the present cross‐sectional data do not isolate independent effects. Knowledge can both influence and result from vaccination contact, and reported barriers may vary with prior service use. Effect estimates and precision should therefore take priority over binary significance labels [5, 16, 17].

Healthcare‐provider information remained positively associated with self‐reported receipt of at least three doses after parity adjustment. This is consistent with evidence that health‐service contact and information exposure are associated with tetanus vaccination [5, 18]. The association does not establish that counseling caused uptake because women with more vaccination contacts may also have received more provider information. Nevertheless, standardized counseling and routine dose‐history checks are reasonable programmatic strategies.

Family and friends were the most frequently reported usual source of information, exceeding healthcare providers. This suggests that vaccination information circulates strongly through social networks in Ifakara. Such networks may reinforce acceptance when messages are accurate, but they may also transmit incomplete or inconsistent information. Rather than treating community communication as separate from facility‐based counseling, maternal immunization programs could align provider messages with community outreach, women's groups, and other trusted local channels. Comparisons with settings where health providers are the dominant information source should be made cautiously because health‐system organization, media exposure, and community structures differ between countries [11].

Access barriers were reported by more than one‐third of participants. Perceived cost was the most frequently selected barrier, followed by lack of information, transportation, and fear of side effects. The questionnaire did not distinguish vaccine price from indirect costs such as transport or time away from work, and the adjusted CI crossed 1 after parity was included. Tanzanian studies have likewise identified transport, distance, information, and service‐delivery challenges [8, 9, 19].

The counterintuitive education finding in the reduced model was explained largely by covariate composition. The secondary‐education group contained more younger and nulliparous women than the primary/no‐formal group, and both education estimates moved toward the null after parity was added. The final model therefore provides no evidence that higher education reduces vaccination. More broadly, parity was the strongest correlate, plausibly reflecting cumulative pregnancy‐related vaccination opportunities; its large OR should not be interpreted as a causal biological effect [7, 17].

The findings have several practical implications. Monitoring should distinguish initial vaccine contact from higher recalled dose thresholds and should verify previous doses where records are available. Younger women and women outside antenatal care should be included in communication and outreach. Healthcare providers may benefit from standardized counseling tools, while community channels remain important because family and friends were prominent information sources.

This study has several limitations. Vaccination history was self‐reported and was not verified with cards or facility records; recall error may increase across several years. The cross‐sectional design prevents causal inference and permits reverse causation among uptake, knowledge, provider information, and barriers. Composite scoring was reconstructed for revision; the attitude scale had modest internal consistency, and questionnaire response options were incomplete for dose knowledge and ambiguous for the highest dose and parity categories. The surviving records did not preserve complete recruitment‐flow counts, a separate interview‐date variable, or sampling‐unit identifiers. Consequently, the exact interview period and response fraction could not be verified, and standard errors could not be adjusted for the multistage design. The documentation also did not separately specify guardian permission or assent procedures for participants aged 16–17 years. One record with missing parity was excluded from the main model. Wide CIs, residual confounding by pregnancy and service contact, and recruitment from one semi‐urban town further limit precision and generalizability.

Despite these limitations, the study provides local evidence that high awareness and initial vaccine contact do not necessarily correspond to higher recalled dose thresholds. The results support better documentation and dose‐history review, provider‐led and community communication, and removal of access barriers, while avoiding inference about verified individual protection from self‐reported dose counts alone.

## 5 Conclusion

Among 383 women aged 15–44 years in Ifakara Town, self‐reported tetanus vaccination uptake was high for ever receiving the vaccine and for at least two or three doses, but lower at four‐ and five‐dose thresholds. In the parity‐adjusted analysis, older age, healthcare‐provider information, and parous status were associated with the ≥ 3‐dose outcome; education, knowledge, and barriers were not independently associated. These non‐causal associations probably reflect, in part, cumulative vaccination opportunities. Strengthening dose‐history review, documentation, provider counseling, community communication, and practical access support remains warranted.

## Author Contributions

**Dorothea R. Mhina:** conceptualization, methodology, supervision, writing – review and editing. **Zacharia L. Laizer:** methodology, formal analysis, writing – review and editing. **Onesmo Tisiani Hilari:** conceptualization, methodology, data curation, investigation, formal analysis, writing – original draft, writing – review and editing. **Byamungu Zephrine Petro:** methodology, writing – review and editing, visualization. **Samweli Y. Bahati:** methodology, data curation, validation, formal analysis, visualization, writing – review and editing, writing – original draft. **Reuben S. Maghembe:** conceptualization, methodology, supervision, funding acquisition, project administration, resources, writing – original draft, writing – review and editing, investigation.

## Funding

The authors have nothing to report.

## Ethics Statement

Ethical approval was granted by the SFUCHAS Research Ethics Review Committee (reference SFU/Re. Pub/Eth. App/Vol.1/4; September 2, 2025) and was valid for 1 year. Ifakara Town Council granted research permission (reference IFTC/E.10/80 VOL V/118; September 12, 2025).

## Consent

Written informed consent was recorded for all participants before data collection. The approved study population included women aged 15–44 years; however, the surviving documentation does not separately specify guardian permission, adolescent assent, an ethics waiver, or an emancipated‐minor provision for participants aged 16–17 years.

## Conflicts of Interest

The authors declare no conflicts of interest.

## Artificial Intelligence Disclosure

During revision, the authors used OpenAI ChatGPT for language editing. The tool was not listed as an author, and the authors reviewed the output and take full responsibility for the manuscript.

## Author Approval and Guarantor Statement

All authors have read and approved the final version of the manuscript. Reuben S. Maghembe, the manuscript guarantor, had full access to all of the data in this study and takes complete responsibility for the integrity of the data and the accuracy of the data analysis.

## Image Permissions

Figures 1 and 2 were created by the authors from the study results and do not reproduce or adapt previously published images.

## Transparency Statement

Reuben S. Maghembe, the manuscript guarantor, affirms that this manuscript is an honest, accurate, and transparent account of the study being reported; that no important aspects of the study have been omitted; and that any discrepancies from the study as planned (and, if relevant, registered) have been explained. The manuscript guarantor attests that all listed authors meet authorship criteria and that no others meeting the criteria have been omitted.

## Supporting information


Supporting File 1

Supporting File 2

## Data Availability

The deidentified participant‐level data are not publicly available because the consent materials did not include public data sharing and the dataset contains combinations of demographic characteristics that may create a risk of re‐identification. Data may be available from Reuben S. Maghembe, the corresponding author (rmaghembe@sfuchas. ac. tz), upon reasonable request and with approval from the SFUCHAS Research Ethics Review Committee.
