# Supplementary material for: Knowledge, Attitudes, and Self‐Reported Tetanus‐Toxoid‐Containing Vaccine Uptake Among Women Aged 15–44 Years in Ifakara Town, Tanzania: A Community‐Based Cross‐Sectional Study
Source: Health Sci Rep. 2026 Sep 14;9(9):e73262. doi: 10.1002/hsr2.73262 (PMC13572905; doi:10.1002/hsr2.73262)
Supplement: Supplementary file 1 [file HSR2-9-e73262-s002.pdf]

Supporting file 1. Study questionnaire

Knowledge, attitudes, and self-reported tetanus-toxoid-containing vaccine uptake among women aged 15–44 years in Ifakara Town, Tanzania: a community-based cross-sectional study

Note: This questionnaire is reproduced from the signed study report for transparency and reproducibility. The question wording and response options are retained, with only minor typographical and formatting standardization.

Section 1. Demographic information

- **Age**
  Response options: ____ years
- **Parity**
  Response options: 0; 1; 2; 3; More than 4
- **Marital status**
  Response options: Single; Married; Divorced/Widowed
- **Education level**
  Response options: No formal education; Primary education; Secondary education; Tertiary education
- **Occupation**
  Response options: Unemployed; Employed (Formal); Self-employed; Student; Peasant

Section 2. Knowledge about tetanus toxoid vaccine

- **KN01. Have you heard of the Tetanus Toxoid Vaccine (TT)?**
  Response options: Yes; No
- **KN02. What is the primary purpose of the TT vaccine? Select all that apply.**
  Response options: To prevent tetanus; To prevent neonatal tetanus; To improve overall health; Other
- **KN03. How many doses of the TT vaccine are recommended for full protection?**
  Response options: 1; 2; 3; Unsure
- **KN04. When should women start receiving TT vaccine, and is it safe during pregnancy?**
  Response options: During adolescence; During pregnancy (safe); Unsure
- **KN05. Where did you obtain your information about the TT vaccine? Select all that apply.**
  Response options: Healthcare provider; Family/Friends; Media (TV, Radio, Internet); Community health programs; Other
- **KN06. How effective do you believe the TT vaccine is in preventing tetanus?**
  Response options: Very effective; Somewhat effective; Not effective; Unsure
- **KN07. How confident are you in your knowledge about the TT vaccine?**
  Response options: Very confident; Somewhat confident; Not confident

Section 3. Attitudes toward vaccination

- **Instruction**
  Response options: Indicate your level of agreement using: 1 = Strongly disagree; 2 = Disagree; 3 = Agree; 4 = Strongly agree
- **AT01. Vaccination is important for your health.**
  Response options: 1; 2; 3; 4
- **AT02. I believe that the TT vaccine is effective in preventing tetanus.**
  Response options: 1; 2; 3; 4
- **AT03. I trust healthcare providers to give accurate information about vaccines.**
  Response options: 1; 2; 3; 4

- **AT04. I feel confident in my ability to make informed decisions about vaccinations.**
  Response options: 1; 2; 3; 4
- **AT05. I would feel safe receiving the TT vaccine.**
  Response options: 1; 2; 3; 4
- **AT06. I am concerned about potential side effects of the TT vaccine.**
  Response options: 1; 2; 3; 4
- **AT07. I believe that getting vaccinated protects not only me but also my community.**
  Response options: 1; 2; 3; 4
- **AT08. I would recommend the TT vaccine to family and friends.**
  Response options: 1; 2; 3; 4
- **AT09. I think cultural beliefs play a significant role in vaccination decisions.**
  Response options: 1; 2; 3; 4
- **AT10. I think that government campaigns effectively promote vaccination.**
  Response options: 1; 2; 3; 4

Section 4. Practices related to tetanus toxoid vaccine utilization

- **PT01. Have you ever received the Tetanus Toxoid Vaccine?**
  Response options: Yes; No
- **PT02. If yes, how many doses of the TT vaccine have you received?**
  Response options: 1; 2; 3; 4; 5
- **PT03. When was the last time you received the TT vaccine?**
  Response options: Within the last year; 1–5 years ago; More than 5 years ago; Never received
- **PT04. Where did you receive the TT vaccine? Select all that apply.**
  Response options: Public health facility; Private clinic; Hospital; Community health program; Other
- **PT05. How do you usually get information about the TT vaccine? Select all that apply.**
  Response options: Healthcare provider; Family/Friends; Media (TV, Radio, Internet); Community health programs; Other
- **PT06. Have you ever faced any barriers in accessing the TT vaccine?**
  Response options: Yes; No
- **PT07. If yes, what barriers did you face? Select all that apply.**
  Response options: Lack of transportation; Cost of the vaccine; Lack of information; Fear of side effects; Other
- **PT08. How likely are you to get the TT vaccine in the future?**
  Response options: Very likely; Likely; Unsure; Unlikely; Very unlikely
